# D-π-A-Type Pyrazolo[1,5-*a*]pyrimidine-Based Hole-Transporting Materials for Perovskite Solar Cells: Effect of the Functionalization Position

**DOI:** 10.3390/ma15227992

**Published:** 2022-11-11

**Authors:** Fatiha Bouihi, Bruno Schmaltz, Fabrice Mathevet, David Kreher, Jérôme Faure-Vincent, Ceren Yildirim, Ahmed Elhakmaoui, Johann Bouclé, Mohamed Akssira, François Tran-Van, Mohamed Abarbri

**Affiliations:** 1 Laboratoire de Physico-Chimie des Matériaux et des Electrolytes pour l’Energie (EA 6299), Université de Tours, Parc de Grandmont, 37200 Tours, France; 2 Laboratoire de Chimie Physique et Biotechnologies des Biomolécules et des Matériaux (LCP2BM), Faculté des Sciences et Techniques de Mohammedia, Université Hassan II de Casablanca, BP 146, Mohammedia 28800, Morocco; 3 Center for Organic Photonics and Electronics Research (OPERA), Kyushu University, 744 Motooka, Nishi-ku, Fukuoka 819-0395, Japan; 4 CNRS, Institut Parisien de Chimie Moléculaire, IPCM, Sorbonne Université, 4 Place Jussieu, 75005 Paris, France; 5 Institut Lavoisier de Versailles, UMR 8180, Université de Versailles Saint-Quentin-en-Yvelines, 78035 Versailles, France; 6 CEA, CNRS, IRIG-SyMMES, Université Grenoble-Alpes, 38000 Grenoble, France; 7 CNRS, XLIM, UMR 7252, Université de Limoges, 87000 Limoges, France

**Keywords:** D-π-A, pyrazolo[1,5-a]pyrimidine, 3,6-CzDMPA, hole-transporting materials, perovskite solar cells

## Abstract

Donor–acceptor (D–A) small molecules are regarded as promising hole-transporting materials for perovskite solar cells (PSCs) due to their tunable optoelectronic properties. This paper reports the design, synthesis and characterization of three novel isomeric D-π-A small molecules **PY1**, **PY2** and **PY3**. The chemical structures of the molecules consist of a pyrazolo[1,5-*a*]pyrimidine acceptor core functionalized with one 3,6-bis(4,4′-dimethoxydiphenylamino)carbazole (3,6-CzDMPA) donor moiety via a phenyl π-spacer at the 3, 5 and 7 positions, respectively. The isolated compounds possess suitable energy levels, sufficient thermal stability (T_d_ > 400 °C), molecular glass behavior with T_g_ values in the range of 127–136 °C slightly higher than that of the reference material Spiro-OMeTAD (126 °C) and acceptable hydrophobicity. Undoped **PY1** demonstrates the highest hole mobility (3 × 10^−6^ cm^2^ V^−1^ s^−1^) compared to **PY2** and **PY3** (1.3 × 10^−6^ cm^2^ V^−1^ s^−1^). The whole isomers were incorporated as doped HTMs in planar *n-i-p* PSCs based on double cation perovskite FA_0.85_Cs_0.15_Pb(I_0.85_Br_0.15_)_3_. The non-optimized device fabricated using **PY1** exhibited a power conversion efficiency (PCE) of 12.41%, similar to that obtained using the reference, Spiro-OMeTAD, which demonstrated a maximum PCE of 12.58% under the same conditions. The **PY2** and **PY3** materials demonstrated slightly lower performance in device configuration, with relatively moderate PCEs of 10.21% and 10.82%, respectively, and slight hysteresis behavior (−0.01 and 0.02). The preliminary stability testing of PSCs is also described. The **PY1-**based device exhibited better stability than the device using Spiro-OMeTAD, which could be related to its slightly superior hydrophobic character preventing water diffusion into the perovskite layer.

## 1. Introduction

Perovskite solar cells (PSCs) are promising and low-cost candidates for photovoltaic technology. Their power conversion efficiency (PCE) has improved significantly, from 3.8% in 2009 to 25.7% currently [1,2]. These significant advances are primarily attributed to the excellent optoelectronic properties of hybrid perovskite materials, such as widely adjustable band gaps, high absorption coefficients, high charge mobilities, long carrier lifetimes and long charge diffusion lengths [3,4]. Despite their high efficiency, several issues such as stability [5], J-V (current density–voltage) hysteresis [6] and lead toxicity [7] still need to be improved for large-scale applications.

In conventional PSC structures, hole-transporting material (HTM) is a key component to achieve high-performance and stable devices, and to reduce hysteresis [8,9]. Its role consists of extracting and transporting photogenerated holes, blocking electrons and protecting the perovskite active layer from external stress such as moisture, oxygen and heat [8,10]. The chemical structure of the HTM and particularly the functional groups can also play a crucial role in the passivation of defects at the perovskite/HTM interface [11,12,13]. An ideal HTM should present suitable energy levels of the highest occupied molecular orbital (HOMO) and the lowest unoccupied molecular orbital (LUMO), high hole mobility, good solubility in common organic solvents and high chemical, thermal, morphological, light and moisture stability [14]. To date, 2,2′,7,7′-Tetrakis[*N*,*N*-di(4-methoxyphenyl)amino]-9,9′-spirobifluorene (Spiro-OMeTAD) is the best-selling HTM in PSCs [15]. However, its synthetic route and purification method remain delicate and expensive [16], and doping strategies induce instability issues [17]. A large number of new p-type organic semiconductor materials based on π-conjugated small molecules and polymers are still to be developed as alternatives [18,19,20,21]. Compared to polymers, π-conjugated small molecules have the advantages of well-defined structures, controlled molecular weight, high purity, good solubility and easy synthesis [22].

Small molecules with a donor–π–acceptor (D-π-A) structure, where the electron donor and electron acceptor moieties are connected via a conjugated π-bridge, have already been employed as an effective approach to design promising HTMs with tunable energy levels and high hole mobility [23,24,25,26]. D-π-A molecular backbones generally exhibit an intramolecular charge transfer (ICT) character, allowing charge separation and strong dipole–dipole interactions, which can induce a specific molecular packaging [27,28]. The charge separation and dipole–dipole interactions could improve the overall charge transport properties [27].

In this context, we develop new, promising D-π-A HTMs for PSC applications. Most research on this type of structure typically focuses on modifying the nature of the donor and acceptor moieties or the π-spacer [23,24,26,29]. However, little attention has been focused on the effect of the functionalization position of the electron acceptor moiety with the electron donor moiety on the physicochemical and photovoltaic properties of the target HTMs.

Therefore, we present the synthesis and characterization of three novel positional isomeric D-π-A type molecules, **PY1**, **PY2** and **PY3**, as HTMs for *n-i-p*-type PSCs. In these molecular structures, the acceptor unit has been functionalized with the donor group via a phenyl π-spacer at different positions to investigate their structure–property relationships. The chosen donor moiety is 3,6-bis(4,4′-dimethoxydiphenylamino)carbazole (3,6-CzDMPA) [30]. 3,6-CzDMPA-based HTMs have attracted much attention due to their interesting physicochemical properties, such as molecular glass behavior, excellent thermal stability and good solubility, and good charge transport properties [31,32]. We have extensively studied this class of HTMs in dye-sensitized solar cells (DSSCs) and PSC applications [11,33,34,35,36,37,38]. It has been demonstrated that the terminal methoxy groups can act as a Lewis base to passivate the defect sites at the perovskite/HTM interface [11,31]. The rigid and planar *N*-heterocyclic pyrazolo[1,5-*a*]pyrimidine system was chosen as the electron acceptor unit [39]. Although this moiety has been widely used to develop an extensive range of fluorescent materials for several opto-electronics applications such as organic light-emitting diodes (OLEDs) [40] and fluorescent sensors [41], we propose for the first time, to our knowledge, its use as HTM in PSCs.

Based on a pyrazolo[1,5-*a*]pyrimidine acceptor core, **PY1**, **PY2** and **PY3** have been functionalized with a 3,6-CzDMPA donor moiety via a phenyl π-spacer at positions 3, 5 and 7, respectively. Their electronic, optical, electrochemical, thermal, hydrophobic and charge transport properties were measured and discussed according to the position of functionalization. The three materials were subsequently doped with 4-*tert*-butylpyridine (*t*-BP) and lithium bis(trifluoromethylsulfonyl)-imide (Li-TFSI) for use as HTMs in planar *n-i-p* FA_0.85_Cs_0.15_Pb(I_0.85_Br_0.15_)_3_-based PSCs [42], and the impact of the functionalization position on the device’s photovoltaic parameters and stability was evaluated.

## 2. Materials and Methods

### 2.1. Materials

3-aminopyrazole, 3-amino-5-phenylpyrazole, 9*H*-carbazole, ethyl phenylpropiolate, 4,4-dimethoxydiphenylamine, 1,3-diphenyl-1,3-propanedione, ethyl benzoylacetate, tri-*tert*-butylphosphine (P(*t*-Bu)_3_), palladium acetate (Pd(OAc)_2_), bis(pinacoloto)diboron, 1,1′-bis(diphenylphosphino)ferrocene]dichloropalladium(II) (Pd(dppf)Cl_2_), sodium hydride (NaH), *N*-bromosuccinimide (NBS), phosphorus(V) oxychloride (POCl_3_), benzyl bromide, potassium iodate (KIO_3_), sodium carbonate (Na_2_CO_3_), potassium iodide (KI), potassium *tert*-butoxide (*t*-BuOK), 1,4-dibromobenzene, *N*,*N*-dimethylaniline, potassium phosphate tribasic (K_3_PO_4_), copper(I) iodide (CuI), *trans*-1,2-diaminecyclohexane, potassium acetate (KoAC), Spiro-OMeTAD, formamidinium iodide (FAI), cesium iodide (CsI), lead iodide (PbI_2_), lead bromide (PbBr_2_), *N*,*N*-dimethylformamide (DMF), tin(IV) oxide (SnO_2_), magnesium sulfate (MgSO_4_), tetraethylammonium tetrafluoroborate (Et_4_NBF_4_), ferrocene, silica gel (SiO_2_), dichloromethane (DCM), chloroform (CHCl_3_), acetic acid (AcOH), dimethyl sulfoxide (DMSO), 1,4-dioxane, tetrahydrofuran (THF), toluene and ethanol (EtOH) were purchased from Sigma-Aldrich, Alfa Aesar or Fluorochem and used as received without any further purification.

### 2.2. Synthesis of New HTMs PY1–PY3

Pyrazolo[1,5-*a*]pyrimidines precursors **2a**–**c** and boronic acid ester **6** were synthesized according to the procedure described in the literature (Figures S1 and S2) [40,43,44,45,46,47,48,49,50].

Corresponding precursor **2a**–**c** (1 equiv), compound **6** (1.1 equiv), Pd(dppf)Cl_2_ (0.1 equiv) and Na_2_CO_3_ (5 equiv) were combined in a microwave tube. A mixture of (5:1) 1,4-dioxane/water was added, and the system was degassed with argon. The reaction was heated in a microwave at 110 °C for 2 h. After completion of the reaction, the residue was purified via silica gel chromatography in petroleum ether/ethyl acetate (8/2) to give the appropriate HTM. Details of structural characteristics (^1^H/^13^C NMR spectroscopy and mass spectrometry) of **PY1**–**PY3** are given in the Supplementary Information (Figures S3–S5).

### 2.3. Devices Fabrication Procedure

Hole mobility of the undoped HTMs was measured by organic field-effect transistors (OFETs) fabricated using a bottom-gate/bottom-contact architecture. The OFET chips were purchased from Fraunhofer IPMS in Dresden, Germany. Briefly, the structures were fabricated on highly doped silicon wafers with thermal SiO_2_ acting as a dielectric and two interdigitated gold electrodes acting as source and drain. Thin film deposition was performed by drop casting a dilute HTM solution in chlorobenzene (10 mg/mL) on pre-patterned substrates. The back gate was realized by contacting the doped silicon on a copper plate by using silver paste. Reported mobility values correspond to a source–drain voltage of −80 V (device in the saturation regime) and for a source–drain electrode spacing of 20 µm. The channel length was 20 µm and the channel width was 10 mm. More details on the OFET structure and electrical measurements can be found in reference [35].

Solar cells were fabricated using the conventional device structure of glass/fluorine-doped tin oxide (FTO)/electron-transporting layer (ETL)/perovskite absorber layer/hole-transporting layer (HTL)/metal electrode (Au).

**ETL.** First, FTO glass substrates were etched using zinc powder and a concentrated hydrochloric acid solution. The substrates were then cleaned sequentially by ultrasonication in deionized water, acetone, isopropanol and ethanol for 15 min in each solvent. After drying with N_2_, the substrates were treated under ultraviolet ozone for 20 min to remove the different contaminants before depositing the ETL. The tin oxide (SnO_2_) layer was then deposited onto the FTO substrates by spin-coating at 4000 rpm for 30 s from 1 mL of a colloidal solution of SnO_2_ (40 wt % suspension in H_2_O) diluted in 10 µL of isopropanol, stirred for 1 h at room temperature. The FTO/SnO_2_ substrates were annealed at 100 °C for 5 min, then at 150 °C for 30 min before treatment with ultraviolet ozone for 20 min.

**Perovskite absorber layer.** FA_0.85_Cs_0.15_Pb(I_0.85_Br_0.15_)_3_ perovskite solution was prepared in a glovebox by mixing four precursors, formamidinium iodide (FAI, 146.3 mg), cesium iodide (CsI, 39 mg), lead iodide (PbI_2_, 392 mg) and lead bromide (PbBr_2_, 55.4 mg), in 600 µL of anhydrous DMF and 78 µL of anhydrous DMSO. The solution was stirred continuously for 3 h at 65 °C, then filtered using a 0.45 µm (PTFE) filter before deposition onto the FTO/SnO_2_ substrates. The perovskite precursor solution (30 µL) was deposited by spin-coating (at 4000 rpm for 30 s) and dripping diethyl ether as an anti-solvent (500 µL) after 10 s of spin-coating. All the films were annealed at 100 °C for 5 min, then at 150 °C for 15 min.

**HTL.** The synthesized HTMs and Spiro-OMeTAD were dissolved in chlorobenzene (30 mg/mL), and 11.8 µL of *t*-BP and 7.2 µL of Li-TFSI (1.8 M solution in acetonitrile) were added as dopants. The solution was stirred continuously at room temperature for 2 to 3 days, then filtered using a 0.45 µm (PTFE) filter before use. The final HTM solution (25 µL) was spin-coated onto the perovskite layers at 3000 rpm (**PY1** and Spiro-OMeTAD) or 2000 rpm (**PY2** and **PY3**) for 30 s. No heating or annealing was required following the deposition.

**Electrode deposition (Au).** After HTL deposition, 100 nm of gold electrodes were thermally evaporated and deposited under a vacuum of 10^−6^ mbar. The active area of the cell was 0.20 cm^2^.

### 2.4. Instrumentation

^1^H and ^13^C NMR spectra were recorded on a Bruker Avance FT-NMR-300 (^1^H: 300 Hz ^13^C: 75 Hz), in DMSO-d_6_ and/or CDCl_3_ as a solvent with TMS as an internal standard. J (coupling constant) values are estimated in Hertz (Hz) and chemical shifts were recorded in ppm on δ scale. The signal patterns are indicated as follows: s, singlet; d, doublet; t, triplet; q, quartet; m, multiplet. Mass spectra (MS) were recorded on a hybrid tandem quadrupole/time-of-flight (Q-TOF) instrument, equipped with a pneumatically assisted electrospray (Z-spray) ion source (Micromass, Manchester, UK) operated in positive mode. Melting temperatures of synthesized compounds were measured on a Kofler block.

UV–visible absorption spectra were recorded with a Jasco V-670 spectrometer in the wavelength range from 250 to 700 nm. Cyclic voltammetry (CV) was performed on a Biologic Applied Research MPG2 multi-channel potentiostat, in solution (5 × 10^−3^ M) with DCM/Et_4_NBF_4_ (0.1 M) as an electrolyte at a scan rate of 5 mV s^−1^, and using platinum (Pt) as a working electrode, silver as a counter and pseudo reference electrode, and the Ferrocene/Ferrocenium redox couple as an internal reference. Differential scanning calorimetry (DSC) measurements were performed on a PerkinElmer DSC-7 (heating/cooling rate 5 °C/min). Thermogravimetric analysis (TGA) was fulfilled using a PerkinElmer TAS-409 thermogravimeter at a heating rate of 10 °C/min under N_2_. Water contact angle (CA) measurements were performed using a goniometer Data-Physics Contact Angle System OCA and analyzed by the SCA20 software through the sessile drop method. Film thicknesses were measured using a profilometer (Dektak-XT, Bruker, Billerica, MA, USA) with a 12.5 µm radius stylus and a stylus force of 1 mg. Current–voltage (J–V) characteristics of the PSCs were measured in the dark and under simulated AM1.5G (100 mW cm^−2^) illumination in ambient conditions using a Keithley 2440 source meter. The light intensity of the simulator was calibrated using a standard silicon solar cell. Forward and backward scans were carried out between 1.5 V and −1 V. The average values of photovoltaic performance were calculated from two devices characterized. Incident photon-to-current efficiency (IPCE) measurements were performed over a wavelength range of 300–900 nm in DC mode on a quantum efficiency system (QE-R, Enli Technology Co., Ltd., Milpitas, CA, USA), calibrated by the silicon reference solar cell.

## 3. Results and Discussion

### 3.1. Design and Synthesis

**Figure 1:**
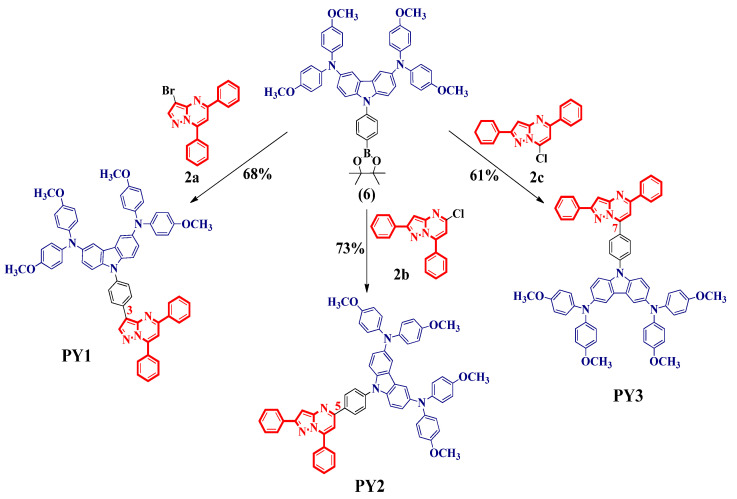
Synthetic route of **PY1**–**PY3** molecules by Suzuki–Miyaura cross-coupling reaction. Reagents and conditions: **2a**–**c** (1 equiv), compound **6** (1.1 equiv), PddppfCl_2_ (0.1 equiv), Na_2_CO_3_ (5 equiv), 1,4-dioxane/water (5:1) under MW irradiation at 110 °C for 2 h.

In this study, the fused pyrazolo[1,5-*a*]pyrimidine system containing five- and six-membered rings (pyrazole and pyrimidine) was selected as an electron acceptor core due to its rigid and planar molecular structure [39], electron-accepting ability [41,51], excellent photostability [52], efficient synthetic approaches [39] and easy functionalization [39,46,53]. The 3,6-bis(4,4′-dimethoxydiphenylamino)carbazole (3,6-CzDMPA) unit was used as an electron donor due to its hole-transporting properties, molecular glass behavior, thermal stability, good solubility and facile synthetic route [30,31]. In addition, the terminal methoxy (-OCH_3_) groups can serve as Lewis base sites to passivate defects at the perovskite/HTM interface [11]. These functional groups can also enhance the electron donor effect from the donor group to the acceptor unit [54]. The phenyl spacer group was incorporated as a π-spacer to extend the π-conjugation of the HTM. Thus, the 3,6-CzDMPA group was covalently bonded to the pyrazolo[1,5-*a*]pyrimidine core via the phenyl π-spacer at position 3 of the fused pyrazole ring and at positions 5 and 7 of the fused pyrimidine ring to obtain three isomers, **PY1**, **PY2** and **PY3**, respectively (Figure 1).

The three novel D-π-A structures, **PY1**–**PY3**, were prepared in a one-step reaction by the Suzuki–Miyaura cross-coupling reaction of the brominated or chlorinated pyrazolo[1,5-*a*]pyrimidine cores **2a**–**c** with the boronic acid ester derived from the 3,6-CzDMPA donor group **6**, using a PddppfCl_2_ catalyst in 1,4-dioxane/water with Na_2_CO_3_ as the base, at 110 °C under microwave (MW) irradiation for 2 h. The three compounds were obtained in yields of 68%, 73% and 61%, respectively. It should be noted that the boronic acid ester **6** was synthesized in six steps, starting from the 9*H*-carbazole, with an overall yield of 43% (Figure S2), higher than the 32% obtained using another reported strategy [55]. The three novel derivatives demonstrate good solubility in common organic solvents, such as toluene and chlorobenzene, typically used for HTM layer deposition in PSCs [56].

### 3.2. Optical, Electrochemical, Thermal and Photophysical Properties

**Figure 2:**
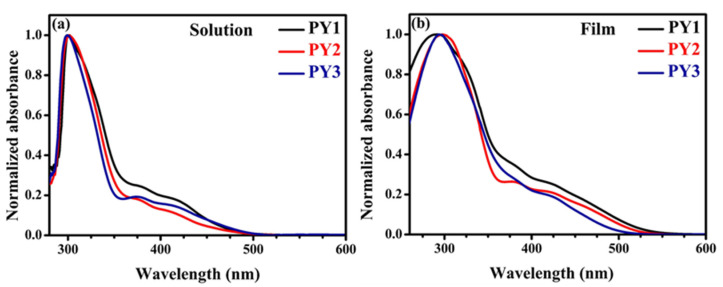
Normalized UV–visible absorption spectra in THF (**a**) and in thin film (**b**) of **PY1**–**PY3**.

Figure 2 presents the UV–visible absorption spectra of **PY1**, **PY2** and **PY3** in THF solution (a) and in a solid state (b). In solution, all the molecules exhibit an intense absorption band at 300–305 nm and a less intense second absorption band in the range of 375–425 nm. The short wavelength band corresponds to the π–π* transition of the 3,6-disubstituted carbazole [57]. The other adsorption bands (375–425 nm) could be attributed to the ICT from the electron-rich 3,6-CzDMPA group to the electron-deficient core, as previously described using the 3,6-CzDMPA group with other acceptor moieties [58]. The ICT absorbance is slightly greater for **PY1** than **PY2** and **PY3**, which could be explained by the higher charge transfer in **PY1** due to the superior donor–acceptor interactions [59]. In the solid state, all the molecules display broad peaks absorption, possibly due to intermolecular interactions in the films [60]. The optical band gaps (E_gap_^opt^ = 1240/λ_onset_^film^) of **PY1**, **PY2** and **PY3**, estimated from the onset wavelengths of the film absorption, are 2.25, 2.33 and 2.40 eV, respectively (Table 1).

**Figure 3:**
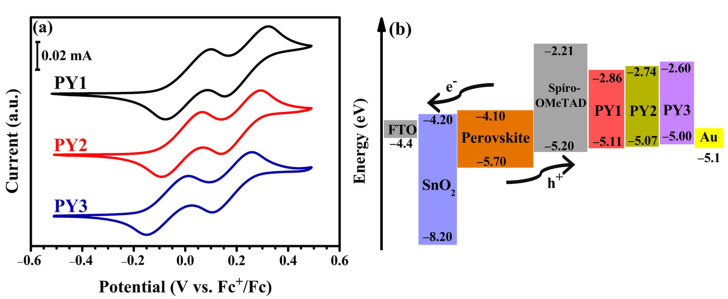
(**a**) CV of **PY1**–**PY3** in DCM (5 × 10^−3^ M); potentials vs. Fc^+^/Fc, 0.1 M Et_4_NBF_4_ at a platinum electrode, scan rate 5 mV s^−1^. (**b**) Energy level diagram of each material in the PSCs.

Figure 3a depicts the cyclic voltammetry (CV) measurements of **PY1**, **PY2** and **PY3** in DCM solution (5 × 10^−3^ M), with 0.1 M of Et_4_NBF_4_ as a supporting electrolyte at 5 mV s^−1^. All the molecules demonstrate two quasi-reversible (see Table 1; ΔE_p_ = 160–180 mV) redox systems, which can be attributed to the formation of the radical cation and dication of the 3,6-disubstituted carbazole units [57]. The disubstitution of carbazole in 3,6-positions with dimethoxy diphenylamine stabilizes the cation radical and the dication by preventing dimer formation [61]. Cyclic voltammetry measurements were carried out for the three compounds to estimate their energy levels (Table 1). The HOMO energy levels were determined from the half-wave potentials (E_1/2_^ox^ = (E_pa_ + E_pc_)/2) of the first oxidation waves. The HOMO values calculated using the equation HOMO = −(eE_1/2_^ox^ + 5.1 eV) [62] were −5.11, −5.07 and −5.00 eV for **PY1**, **PY2** and **PY3**, respectively (vs. −5.20 eV for Spiro-OMeTAD) [37]. The HOMO levels of the three synthesized targets are higher than the valence band of FA_0.85_Cs_0.15_Pb(I_0.85_Br_0.15_)_3_ perovskite (−5.70 eV) used in this study [42], which ensures suitable hole injection from the perovskite layer [14]. The **PY1**, **PY2** and **PY3** LUMO values, calculated from the HOMO and optical bandgap values, are −2.86, −2.74 and −2.60 eV, respectively (vs. −2.21 eV for Spiro-OMeTAD) [37]. Therefore, LUMO values higher than the conduction band of the perovskite layer (−4.1 eV) [42] can effectively block electron transfer to the metal electrode [14]. Figure 3b presents the energy levels of the different layers used in the fabricated PSCs, comparing HTMs, **PY1**, **PY2**, **PY3** and Spiro-OMeTAD.

Regarding the thermal properties of the HTMs, the molecular glass behavior and high thermal stability of the materials are favorable for obtaining suitable organic films [63]. The thermal properties of the new derivatives, investigated using thermogravimetric analysis (TGA) and differential scanning calorimetry (DSC), are presented in Figures S6 and S7 and summarized in Table 1. Thermogravimetric analysis was carried out under a nitrogen atmosphere at a heating rate of 10 °C/min, from 30 °C to 500 °C. The results indicate that all the compounds are thermally stable, with decomposition temperatures (T_d_) (weight loss of 5%) of 410 °C, 430 °C and 440 °C for **PY1**, **PY2** and **PY3**, respectively, which are comparable with that of Spiro-OMeTAD (449 °C) [64].

Differential scanning calorimetry was subsequently performed at heating and cooling rates of 5 °C/min in a nitrogen atmosphere. The second heating DSC curves are given in Figure S6. The results indicate that the three compounds have a stable amorphous morphology, with a high glass transition temperature (T_g_) > 120 °C, which is beneficial for improving device stability [14,65]. While **PY1** exhibits a lower T_g_ of 127 °C, similar to that of Spiro-OMeTAD (126 °C) [64], **PY2** and **PY3** demonstrate slightly higher T_g_ at 136 °C and 135 °C, respectively.

The hole mobility of the HTMs is also a crucial parameter for estimating their potential in PSCs [66]. Hence, the hole mobility of **PY1**, **PY2** and **PY3** was measured using organic field-effect transistor (OFET) devices. All compounds were used without doping. The hole mobility values are given in Table 1. The **PY1** molecule demonstrated a modest hole mobility of 3 × 10^−6^ cm^2^ V^−1^ s^−1^, which is approximately twice as large as that of **PY2** and **PY3** (1.3 × 10^−6^ cm^2^ V^−1^ s^−1^). The higher hole mobility of **PY1** could be related to a superior ICT character, as observed by the UV–visible measurements [67]. These values are around one order of magnitude smaller than Spiro-OMeTAD hole mobility (~2.5 × 10^−5^ cm^2^ V^−1^ s^−1^) obtained under the same conditions (in the saturation regime) [37].

### 3.3. DFT Calculations

**Figure 4:**
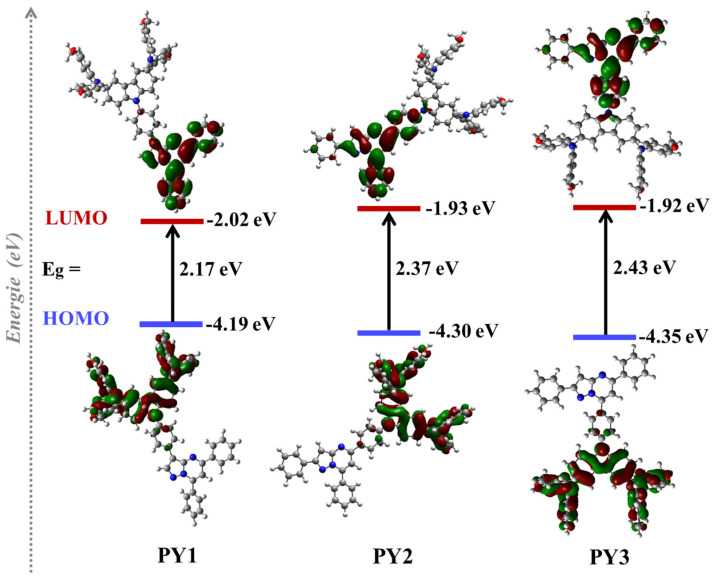
Optimized structures, HOMO and LUMO energy levels, energy gaps, and electron distributions of **PY1**, **PY2** and **PY3** molecules calculated by DFT calculations at the B3LYP/6-31G level.

To investigate the electronic properties of the new compounds, density functional theory (DFT) calculations were performed using the Gaussian 09 program at the B3LYP/6-31G level. The optimized structures, HOMO and LUMO energy levels, energy gaps and electron density distributions of molecules **PY1**–**PY3** are presented in Figure 4.

As illustrated in Figure 4, the frontier molecular orbitals indicate that the **PY1**, **PY2** and **PY3** HOMOs are primarily localized on the 3,6-CzDMPA donor group. This result confirms the electrochemical anodic oxidation of disubstituted carbazole moieties previously obtained by CV [68]. Regarding **PY2** and **PY3**, LUMOs are localized partly on the pyrazolo[1,5-a]pyrimidine acceptor moiety and largely on the phenyl π-spacer. In contrast, the **PY1** LUMO is localized only in the acceptor core, suggesting a stronger acceptor character of pyrazolo[1,5-a]pyrimidine with a substitution in position 3 compared to the other substitutions [69]. In addition, the theoretical calculated HOMO/LUMO values of **PY1**, **PY2** and **PY3** are −4.19/−2.02, −4.30/−1.93 and −4.35/−1.92, respectively, and the energy gap consequently increases in the order of **PY1** (2.17 eV) < **PY2** (2.37 eV) < **PY3** (2.43 eV), following the same trend as the UV–visible characterizations (E_gap_^opt^ = 2.25, 2.33 and 2.40 eV for **PY1**, **PY2** and **PY3**, respectively). These values indicate that the functionalization of the acceptor core at position 3 (**PY1**) decreases the LUMO value, resulting in a smaller band gap. This result is consistent with the stronger acceptor character of the pyrazolo[1,5-a]pyrimidine core [70,71]. Thus, it seems that the ICT between the donor group and the acceptor core could be more significant in the **PY1** molecule than in **PY2** and **PY3** [70], which is in agreement with the UV–visible and hole mobility measurements.

The optimized structures of the three novel molecules have also been calculated employing the DFT method. As depicted in Figure S8, the dihedral angle between the pyrazolo[1,5-a]pyrimidine acceptor core and the phenyl spacer decreases in the order **PY3** (34.64°) > **PY2** (16.45°) > **PY1** (15.88°). This result indicates that the functionalization at position 3 (**PY1**) leads to the smallest twist and, therefore, a potentially better packing [72], which is in accordance with the UV–visible characterizations.

### 3.4. Photovoltaic Performances

The three novel synthesized D-π-A structures were then incorporated as HTMs in planar PSCs with an *n-i-p* structure, glass/FTO/SnO_2_/FA_0.85_Cs_0.15_Pb(I_0.85_Br_0.15_)_3_/HTM/Au (Figure S9) [42], for comparison with a similar Spiro-OMeTAD-based device. Tin oxide (SnO_2_) was used as an electron transport layer (ETL) due to its high electron mobility, wide band gap (~3.6 eV) and good chemical stability [73]. Double cation perovskite, FA_0.85_Cs_0.15_Pb(I_0.85_Br_0.15_)_3_ with an optical bandgap of ~1.60 eV, was chosen as the active layer owing to its superior stability produced by Cs^+^ and Br^-^ [42,74]. The thicknesses of the **PY1**, **PY2** and **PY3** films optimized by controlling the spin-coating speed of HTM solution (30 mg/mL in chlorobenzene) deposition were 96 ± 1, 102 ± 2 and 98 ± 1 nm, respectively (~99 ± 2 nm with Spiro-OMeTAD).

All the HTMs were doped using Li-TFSI (7.2 µL from a 1.8 M solution in acetonitrile) and *t*-BP (11.8 µL). Li-TFSI improves the HTM’s conductivity and, hence, device performance [75]. *t*-BP functions as a morphology controller in the HTM layer by improving Li-TFSI solubility in the HTM solution and, therefore, the quality of the deposited film, which improves the charge extraction at the perovskite/HTM interface [76].

**Figure 5:**
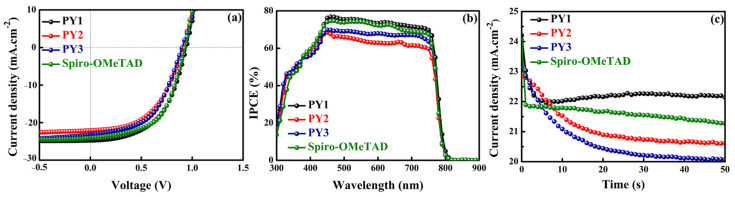
(**a**) Best J-V curves of the champion PSCs based on different doped HTMs measured under simulated AM1.5G illumination; active area = 0.20 cm^2^. (**b**) Corresponding IPCE spectra. (**c**) Stabilized photocurrent density obtained while holding the best PSCs at a bias voltage of 0 V and under continuous illumination.

Figure 5a presents the current density–voltage (J-V) curves of the champion PSCs based on **PY1**, **PY2**, **PY3** and Spiro-OMeTAD measured under AM1.5G (1000 W m^−2^) illumination. Table 2 summarizes the best and average photovoltaic parameter values obtained during forward (FW) and backward (BW) scans of devices using the different HTMs. Perovskite solar cells typically exhibit hysteresis of the J-V curves regarding the scanning direction and speed, particularly in planar architectures [6,77]. This phenomenon can generally be attributed to interfacial charge accumulation, which results from ion migration, high trap density or unbalanced charge carrier transport in PSCs [77,78]. Hence, hysteresis of the champion devices was evaluated using a hysteresis index (HI) defined by the following equation [78]:

HI = (PCE_BW_ − PCE_FW_)/PCE_BW_

As demonstrated in Table 2, small standard deviations were obtained, indicating adequate reproducibility of the photovoltaic performances. The champion device based on **PY1** demonstrated the highest PCE of 12.41% (average of 12.11 ± 0.30%) with a short-circuit current density (J_SC_) of 24.70 mA cm^−2^, an open-circuit voltage (V_OC_) of 0.93 V and a fill factor (FF) of 53% in the BW scan, which is similar to the PCE of 12.58% for the control device using the Spiro-OMeTAD. However, these devices also exhibited hysteresis behavior, with relatively large HIs of 0.15 and 0.17. In contrast, the champion PSCs using **PY2** and **PY3** exhibited low performance, with PCEs of 10.21% (10.11 ± 0.10%) and 10.82% (10.39 ± 0.37%), respectively, and low hysteresis (−0.01 and 0.02). The superior efficiency of **PY1** is primarily attributed to the increased J_SC_ and FF values due to its higher hole mobility (3 × 10^−6^ cm^2^ V^−1^ s^−1^) [79]. It is important to note that our way is in the range of 13–15% PCE because it does not present any specific strategies for defect passivation or use specific additives for crystallization improvement [42,80,81]. The ultimate efficiencies we can achieve in these conditions are lower than the highest reported data [82,83,84]. Nevertheless, we believe that around 13% device efficiency, achieved using our technique, is acceptable and allows us to draw consistent structure–property relationships for this novel class of HTMs.

The results also indicate that the HI value has been reduced in the case of devices using **PY2** and **PY3**. Indeed, the hysteresis behavior of PSCs is affected by multiple factors, including device architecture, properties of the perovskite active layer, nature of the ETL and HTL and their interfaces with the active layer [77,85]. Especially, the influence of the HTL on the J-V curve hysteresis has been revealed in several reports, through the crucial importance of the energetic alignment between the perovskite and the extraction layer, and the associated hole transfer mechanisms [86]. Especially, interfacial defects induced from the solution process, which are mostly HTM-dependent, can lead to variable charge accumulation at the perovskite/HTM interface, resulting in quite different hysteresis behavior of the devices. The charge transport properties of the HTM also play a role in this effect. In our case, the estimated HOMO/LUMO energies and hole mobilities reported in Table 1 for the three compounds suggest that the main origin of the different hysteresis behavior between **PY1** and both **PY2** and **PY3** is mainly associated with the properties of the resulting interface with the perovskite. On the one hand, slightly different trap densities at the interface, induced from the structural variations between the HTM, could lead to slightly enhanced interfacial recombination process in the case of **PY1**. However, this trend is probably not significant considering the V_OC_ values reported for the three devices in Table 2. On the other hand, the specific localization of the LUMO of **PY1** on the acceptor core can be responsible for the different hysteresis behaviors observed with regard to **PY2** and **PY3**.

Figure 5b presents the incident photon-to-current conversion efficiency (IPCE) spectra of the champion PSCs with the three new HTMs and the reference. The devices based on **PY1** and Spiro-OMeTAD afforded a strong spectral response with IPCEs of over 70% compared to **PY2** and **PY3**, in the 450–750 nm wavelength range, suggesting improved hole extraction capability. The integrated Jsc values from the IPCE data were 18.52, 16.06, 17.02 and 18.00 mA.cm^−2^ for the **PY1**, **PY2**, **PY3** and Spiro-OMeTAD-based devices, respectively, indicating similar trends with Jsc obtained from J-V measurements. The moderate deviation observed with the experimental Jsc values (25–27%) can be related to slightly different working conditions used for IPCE measurements in this case (DC mode with no light bias).

For preliminary stability testing, a stabilized current density (J_SC_) measurement was performed by holding the best cells under short-circuit conditions and under continuous illumination (Figure 5c). The **PY1**, **PY2** and **PY3** devices achieved stabilized J_SC_ of 22.20, 20.60 and 20.00 mA cm^−2^, respectively, compared to 21.25 mA.cm^−2^ for the control device. In comparison, the **PY1**-based device exhibited slightly higher stability than the device with Spiro-OMeTAD. These results could be related to the hydrophobic character of HTMs, an important factor for improving device stability by preventing water diffusion into the perovskite layer [57]. To study the hydrophobicity of the three novel molecules compared to Spiro-OMeTAD, water contact angles were measured on HTM films deposited on a glass substrate. As displayed in Figure S11, **PY1**, **PY2**, **PY3** and Spiro-OMeTAD films exhibit water contact angles of 95.0°, 93.8°, 93.4° and 94.5°, respectively, confirming that these four molecules have comparable hydrophobicity but with a slight improvement in the case of **PY1**, in agreement with the preliminary device stability results.

In conclusion, the results indicate that the device using 3-isomer, **PY1**, exhibited an efficiency higher than that of the 5,7-isomer (**PY2** and **PY3**)-based cells. This finding can be attributed to its higher hole-transport capability. In addition, the **PY1**-based device exhibited similar efficiency, but slightly higher stability compared to the device using Spiro-OMeTAD.

## 4. Conclusions and Future Prospects

Three novel D-π-A positional isomers, **PY1**, **PY2** and **PY3**, based on pyrazolo[1,5-*a*]pyrimidine acceptor cores (A) and functionalized by 3,6-CzDMPA donor groups (D) through phenyl π-spacers at positions 3, 5 and 7, respectively, were designed, synthesized and characterized. The physicochemical features reveal that all the target molecules exhibit high thermal stability (T_d_ > 400 °C), molecular glass behavior (T_g_ > 120 °C) and suitable hydrophobic character. These compounds also exhibit suitable frontier energy levels concerning the FA_0.85_Cs_0.15_Pb(I_0.85_Br_0.15_)_3_ perovskite absorber, allowing suitable charge transfer. Moreover, **PY1** with a probable strong ICT character demonstrates the highest hole mobility of 3 × 10^−6^ cm^2^ V^−1^ s^−1^.

These simple molecules were consequently employed as doped HTMs in planar PSCs with an *n-i-p* structure, glass/FTO/SnO_2_/FA_0.85_Cs_0.15_Pb(I_0.85_Br_0.15_)_3_/**HTM**/Au. The device fabricated using **PY1** exhibited the highest PCE (12.41%), with significant hysteresis (0.15). This efficiency is comparable to that of Spiro-OMeTAD (12.58%) under the same conditions. However, the functionalization of the acceptor core at positions 5 and 7 in **PY2** and **PY3** led to lower PCEs of 10.21% and 10.82%, respectively, with considerably less hysteresis (−0.01 and 0.02). Conversely, the preliminary stability test results suggest that the **PY1**-based device exhibits slightly higher stability than the device using the Spiro-OMeTAD. The results highlight the importance of the functionalization position in improving the photovoltaic performance and stability of the target HTM-based devices. In summary, we have demonstrated that **PY1**, bearing the donor group at position 3, is the most promising HTM and remains competitive with Spiro-OMeTAD-based PSCs.

In the future, we will discuss other novel pyrazolo[1,5-*a*]pyrimidine-based D-π-A structures bearing several 3,6-CzDMPA donor moieties and their use as HTMs in PSCs. Theoretical experiments are underway to evaluate their internal hole reorganization energy (λ_h_) [87] and also that of the three HTMs **PY1**–**PY3** discussed in this paper, which will allow us to investigate the structure–hole mobility relationship, including the doping effect on the charge transport properties.

## Figures and Tables

**Table 1:** Optical, electrochemical, thermal and mobility properties of the compounds.

HTM	E_gap_^opt^ (eV) ^1^	E_1/2_^ox^ (V/Fc+:Fc) ^2^	△E_p1_ (V/Fc^+^:Fc) ^3^	△E_p2_ (V/Fc+:Fc) ^4^	E_HOMO_ (eV) ^5^	E_LUMO_ (eV) ^6^	T_g_ (°C) ^7^	T_d_ (°C) ^8^	Hole Mobility (cm^2^ V^−1^ s^−1^) ^9^
PY1	2.25	0.01	0.17	0.17	−5.11	−2.86	127	410	3 × 10^−6^
PY2	2.33	−0.03	0.18	0.18	−5.07	−2.74	136	430	1.3 × 10^−6^
PY3	2.40	−0.10	0.16	0.16	−5.00	−2.60	135	440	1.3 × 10^−6^
Spiro- OMeTAD ^10,11^	2.99	/	/	/	−5.20	−2.21	126	449	2.5 × 10^−5^

^1^ Derived from the onset of absorption (E_gap_^opt^ = 1240/λ_onset_). ^2^ Determined from CV measurements in 0.1 M solution of Et_4_NBF_4_/DCM using Pt working electrode, with a scan rate of 5 mV.s*^−^*^1^ (E_1/2_^ox^ = (E_pa_ + E_pc_)/2, V vs. Fc^+^/Fc). ^3,4^ Potential difference between the cathodic and anodic peak (△E_p_ = E_pc_ − E_pa_, V vs. Fc^+^/Fc). ^5^ Calculated with the following equation: E_HOMO_ = −(eE_1/2_^ox^ + 5.1 eV). ^6^ Estimated by E_LUMO_ = E_HOMO_ + E_gap_^opt^. ^7^ Determined from the second cycle of the DSC (5 °C/min and under N_2_ atmosphere). ^8^ Determined from TGA (10 °C/min under N_2_ atmosphere). ^9^ Measured by OFETs in the saturation regime. ^10,11^ Data from Refs. [37] and [64], respectively.

**Table 2:** Photovoltaic parameters of the PSCs based on different HTMs.

HTM	Scan Direction	J_SC_ (mA cm^−2^)	V_OC_ (V)	FF (%)	PCE (%)	HI
PY1	FW	22.44 ^1^ 22.34 ^2^ ± 0.10 ^3^	0.94 0.93 ± 0.01	49 48 ± 1	10.55 10.31 ± 0.23	0.15
	BW	24.70 24.82 ± 0.12	0.93 0.92 ± 0.01	53 52 ± 1	12.41 12.11 ± 0.30	
PY2	FW	22.05 21.95 ± 0.10	0.90 0.88 ± 0.02	51 52 ± 1	10.21 10.11 ± 0.10	−0.01
	BW	22.90 22.80 ± 0.10	0.84 0.83 ± 0.01	52 53 ± 2	10.10 10.34 ± 0.23	
PY3	FW	22.46 22.34 ± 0.10	0.96 0.95 ± 0.01	49 50 ± 1	10.60 10.74 ± 0.14	0.02
	BW	23.17 22.79 ± 0.37	0.90 0.89 ± 0.01	51 50 ± 2	10.82 10.39 ± 0.37	
Spiro- OMeTAD	FW	23.11 23.02 ± 0.08	0.92 0.93 ± 0.01	48 50 ± 1	10.44 10.84 ± 0.40	0.17
	BW	24.03 23.59 ± 0.43	0.92 0.93 ± 0.02	56 55 ± 1	12.58 12.46 ± 0.20	

^1^ Champion device; ^2^ average on 2 devices; ^3^ standard deviation of the average.

## Data Availability

Not applicable.

## Supplementary Materials

The following supporting information can be downloaded at: https://www.mdpi.com/article/10.3390/ma15227992/s1, S1: Synthetic routes of the acceptor and donor moieties, Figure S1: Synthesis of pyrazolo[1,5-*a*]pyrimidines acceptor cores **2a–c**, Figure S2: Synthesis of compound **6**, S2: Characterization of new compounds, Figure S3: NMR spectrum of **PY1**. (**a**) ^1^H NMR spectrum in DMSO-d_6_ and (**b**) ^13^C NMR spectrum in CDCl_3_/DMSO-d_6_, Figure S4: NMR spectrum of **PY2**. (**a**) ^1^H NMR spectrum and (**b**) ^13^C NMR spectrum in CDCl_3_/DMSO-d_6_, Figure S5: NMR spectrum of **PY3**. (**a**) ^1^H NMR spectrum in DMSO-d_6_ and (**b**) ^13^C NMR spectrum in CDCl_3_/DMSO-d_6_. Figure S6: DSC traces of the three HTMs (second heating) at a scan rate of 5 °C/min under N_2_ atmosphere, Figure S7: TGA curves of the three HTMs at a heating rate of 10 °C/min under N_2_ atmosphere, Figure S8: Geometry optimized structures of **PY1**–**PY3** and dihedral angles between phenyl spacer and pyrazolo[1,5-*a*]pyrimidine acceptor core, calculated by DFT at the B3LYP/6-31G level, Figure S9: Schematic of the device configuration used, Figure S10: J-V curves of the best PSCs using different HTMs under simulated AM1.5G illumination (forward and backward), Figure S11: Water contact angles measurement of the three HTMs and Spiro-OMeTAD films deposited on glass substrates.
