# Supplementary material for: D-π-A-Type Pyrazolo[1,5-a]pyrimidine-Based Hole-Transporting Materials for Perovskite Solar Cells: Effect of the Functionalization Position
Source: Materials (Basel). 2022 Nov 11;15(22):7992. doi: 10.3390/ma15227992 (PMC9697137; doi:10.3390/ma15227992)
Supplement: Supplementary file 1 [file materials-15-07992-s001.zip › materials-2006164-supplementary.pdf]

D-π-A-type pyrazolo[1,5-*a*]pyrimidine-based hole-transporting materials for perovskite solar cells: effect of the functionalization position

Fatiha Bouihi [1,2], Bruno Schmaltz [1,*], Fabrice Mathevet [3,4], David Kreher [4,5], Jérôme Faure-Vincent [6], Ceren Yildirim [7], Ahmed Elhakmaoui [2], Johann Bouclé [7], Mohamed Akssira [2], François Tran-Van [1,*] and Mohamed Abarbri[1,*]

1 Laboratoire de Physico-Chimie des Matériaux et des Electrolytes pour l'Energie (EA 6299), Université de Tours, Parc de Grandmont, 37200 Tours, France
2 Laboratoire de Chimie Physique et Biotechnologies des Biomolécules et des Matériaux (LCP2BM), Faculté des Sciences et Techniques de Mohammedia, Université Hassan II de Casablanca, BP 146, Mohammedia 28800, Maroc
3 Center for Organic Photonics and Electronics Research (OPERA), Kyushu University, 744 Motooka, Nishi-ku, Fukuoka 819-0395, Japan
4 CNRS, Institut Parisien de Chimie Moléculaire, IPCM, Sorbonne Université, 4 Place Jussieu, 75005 Paris, France
5 Institut Lavoisier de Versailles, UMR 8180, Université de Versailles Saint-Quentin-en-Yvelines, France
6 CEA, CNRS, IRIG-SyMMES, Université Grenoble-Alpes, 38000 Grenoble, France
7 CNRS, XLIM, UMR 7252, Université de Limoges, 87000 Limoges, France
* Correspondence : francois.tran@univ-tours.fr (F.T.-V.); mohamed.abarbri@univ-tours.fr (M.A.)

S1. Synthetic routes of the acceptor and donor moieties

The synthetic route to pyrazolo[1,5-*a*]pyrimidine derivatives **2a**–**c**, precursors of the target molecules, is described in Figure S1 [40,43–47]. In a first step, cyclocondensation reactions between 1,3-biselectrophilic substrates, mainly 2-benzoylacetophenone **2**, ethyl phenylpropiolate **3**, or 1-ethoxy-3-phenylpropane-1,3-dione **4**, and the corresponding 3-amino-1*H*-pyrazoles **1** as 1,3-bisnucleophiles, gave compounds **1a**–**c** in 85%, 68%, and 77% yields, respectively. Then, the 3 position of the fused pyrazole ring **1a** was easily functionalized by bromination using *N*-bromosuccinimide (NBS) in acetonitrile, to give the brominated derivative **2a** (70%). The halogenation of **1b** and **1c** bearing an enone substituent at positions 5 and 7 of the fused pyrimidine ring, respectively, was achieved using the usual reagent, phosphorus oxychloride ($POCl_3$), to provide the chlorinated derivatives **2b** (70%) and **2c** (60%).

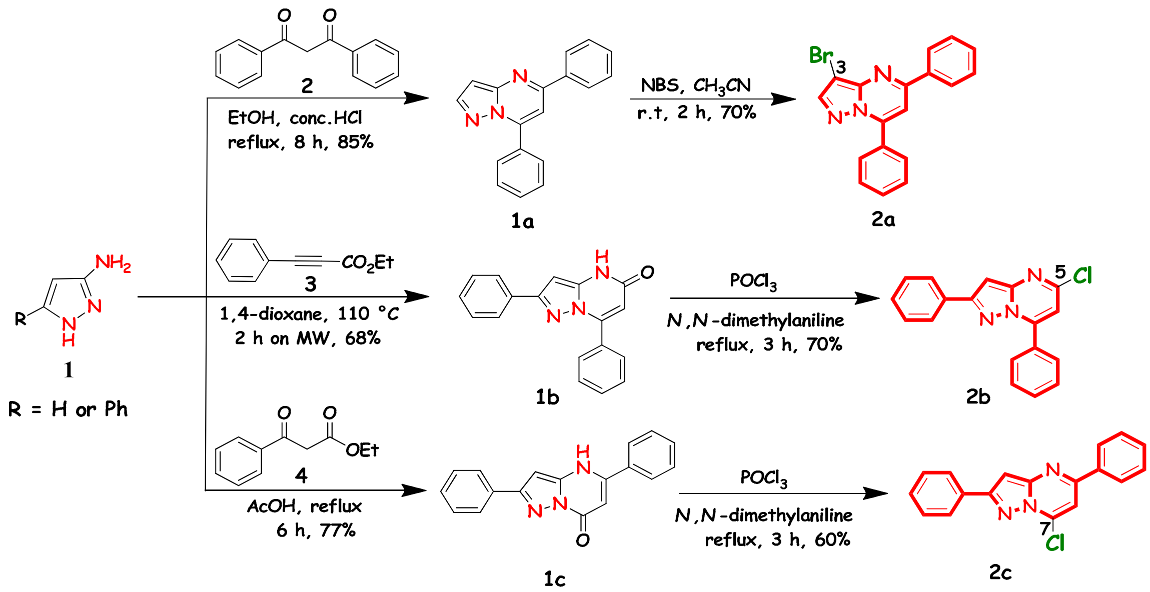

Figure S1. Synthesis of pyrazolo[1,5-*a*]pyrimidines acceptor cores **2a–c**.

Figure S2 depicts the synthesis route of compound **6** that begins with the protection of the carbazole nitrogen using benzyl bromide and NaH in THF at room temperature with a yield of 90% [48]. Then, the iodination of the carbazole in glacial acetic acid at 85°C in the presence of KI/KIO_3 gave 3,6-diiodocarbazole **2** in 95% yield [49]. The following step is a palladium-catalyzed C-N cross-coupling reaction with dimethoxydiphenylamine units, to yield compound **3** (97%) [48]. Deprotection of carbazole nitrogen, using *t*-BuOK in DMSO/THF under oxygen bubbling at room temperature yielded compound **4** (90%) [48]. The *N*-arylation of the latter product using 2 equivalents of 1,4-dibromobenzene was carried out in 1,4-dioxane at 110°C, in the presence of CuI, K_3PO_4, and *trans*-1,2-diaminocyclohexane, to afford compound **5** (70%) [50]. This compound was successfully converted to a boronic acid ester **6** in 82% yield by a Pd-catalyzed Suzuki-Miyaura cross-coupling reaction with bis(pinacolato)diboron [50].

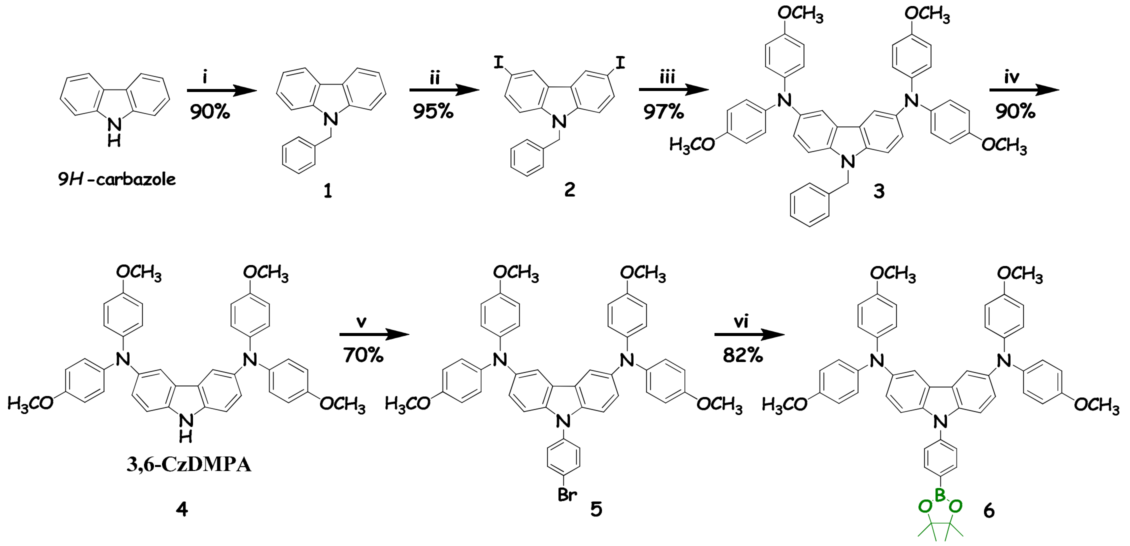

Figure S2. Synthesis of compound **6**. Reagents and conditions: (i) Benzyl bromide (2 equiv), NaH (1.5 equiv), THF, r.t, 6 h; (ii) KI (1.3 equiv), KIO_3 (0.8 equiv), AcOH, 80 °C, 2 h; (iii) Bis(4-methoxyphenyl)-amine (2.2 equiv), $Pd(OAc)_2$ (10 mol%), P(*t*-Bu)$_3$ (20 mol%), *t*-BuOK (3 equiv), toluene, 110 °C, 12 h or 1 h on MW; (iv) O_2, *t*-BuOK (5 equiv), DMSO/THF, r.t, 4 h; (v) 1,4-dibromobenzene (2 equiv), K_3PO_4 (2.5 equiv), CuI (20 mol%), *trans*-1,2-diaminecyclohexane (30 mol%), 1,4-dioxane, 110 °C, 8 h; (vi) Bis(pinacoloto)diboron (1.2 equiv), $PddppfCl_2$ (10 mol%), KoAC (2.2 equiv), 1,4-dioxane, 110 °C, 6 h.

S2. Characterization of new compounds

Compound 2b. Yield (70%); yellow solid; m.p =144–146 °C; R_f = 0.51 (PE/EtOAc, 8:2). ^{1}H NMR (300 MHz, $CDCl_3$, δ): 8.15 (dd, *J* = 7.6, 2.1 Hz, 2H), 7.99 (dd, *J* = 8.1, 1.5 Hz, 2H), 7.65–7.39 (m, 6H), 6.98 (s, 1H), 6.88 (s, 1H). ^{13}C NMR (75 MHz, $CDCl_3$, δ): 156.7 (C), 150.3 (C), 149.9 (C), 147.71 (C), 132.4 (C), 131.6 (C), 130.0 (C), 129.6 (2C), 129.3 (C), 128.7 (2C), 128.6 (2C), 126.7 (2C), 107.5 (C), 93.7 (C). HRMS (ESI) *m*/*z* $[M+H]^+$ calcd for $C_{18}H_{12}ClN_3$: 306.07825; found: 306.07948.

PY1. Yield (68%); yellow solid; m.p = 218–200 °C; R_f = 0.48 (PE/EtOAc, 8:2). ^{1}H NMR (300 MHz, DMSO-*d*$_6$, δ): 8.93 (s, 1H), 8.58 (d, *J* = 7.9 Hz, 2H), 8.46 (d, *J* = 7.9 Hz, 2H), 8.27–8.25 (m, 2H), 7.92 (s, 1H), 7.75 –7.60 (m, 10H), 7.41 (d, *J* = 8.7 Hz, 2H), 7.11 (d, *J* = 8.7 Hz, 2H), 6.91–6.81 (m, 16H), 3.70 (s, 12H). ^{13}C NMR (75 MHz, $CDCl_3$/DMSO-*d*$_6$, δ): 156.3, 154.6 (4C), 147.2, 146.0, 142.9 (4C), 142.5, 141.6, 137.7 (2C), 137.2 (2C), 131.5, 131.3, 131.1, 130.6, 129.3 (2C), 129.0, 128.8 (2C), 127.4 (2C), 127.3 (2C), 127.0 (2C), 124.4 (2C), 124.3 (8C), 123.9 (2C), 116.5 (2C), 114.5 (10C),

110.7 (2C), 109.8, 105.4, 55.5 (4C). HRMS (ESI) *m*/*z* $[M+H]^+$ calcd for $C_{64}H_{50}N_6O_4$: 966.38881; found: 966.39258.

PY2. Yield (73%); yellow solid; m.p = 212–214 °C; R_f = 0.55 (PE/EtOAc, 8:2). ^1H NMR (300 MHz, $CDCl_3$/DMSO-d_6, δ): 8.28 (d, *J* = 8.5 Hz, 2H), 8.18–8.15 (m, 2H), 7.93 (d, *J* = 8.5 Hz, 2H), 7.60 (d, *J* = 9.0 Hz, 2H), 7.55–7.52 (m, 5H), 7.39–7.29 (m, 6H), 7.04 (dd, *J* = 9.0, 1.9 Hz, 2H), 7.01 (s, 1H), 6.88 (d, *J* = 8.8 Hz, 8H), 6.67 (d, *J* = 8.8 Hz, 8H), 3.67 (s, 12H). ^{13}C NMR (75 MHz, $CDCl_3$/DMSO-d_6, δ): 156.0 (4C), 154.8, 151.0, 146.3 (2C), 135.8 (C), 132.9 (4C), 131.3 (3C), 131.1 (2C), 129.6 (8C), 129.0 (2C), 128.8 (2C), 128.8 (5C), 128.6 (10C), 126.5 (5C), 124.3 (2C), 124.2 (2C), 114.6 (2C), 110.7 (2C), 104.8, 93.7, 55.4 (4C). HRMS (ESI) *m*/*z* $[M+H]^+$ calcd for $C_{64}H_{50}N_6O_4$: 966.38881; found: 966.39265.

PY3. Yield (61%); orange solid; m.p = 216–218 °C; R_f = 0.42 (PE/EtOAc, 8:2). ^1H NMR (300 MHz, DMSO-d_6, δ): 8.62 (d, *J* = 8.4 Hz, 2H), 8.35–8.33 (m, 2H), 8.10 (d, *J* = 8.4 Hz, 2H), 7.91 (s, 1H), 7.85 (d, *J* = 8.6 Hz, 2H), 7.69–7.38 (m, 11H), 7.09 (d, *J* = 8.6 Hz, 2H), 6.88–6.78 (m, 16H), 3.68 (s, 12H). ^{13}C NMR (75 MHz, $CDCl_3$/DMSO-d_6, δ): 151.7 (4C), 151.4 (2C), 150.1, 146.5 (4C), 140.6, 137.6, 132.8 (2C), 132.3 (2C), 128.2, 126.4 (2C), 125.6, 124.9 (2C), 124.3 (2C), 124.2 (8C), 124.0 (2C), 122.5 (2C), 121.9 (2C), 121.5, 119.8 (2C), 119.7 (2C), 119.6 (2C), 111.6 (2C), 109.8 (8C), 106.0 (2C), 100.2, 89.2, 50.8 (4C). HRMS (ESI) *m*/*z* $[M+H]^+$ calcd for $C_{64}H_{50}N_6O_4$: 966.38881; found: 966.39244.

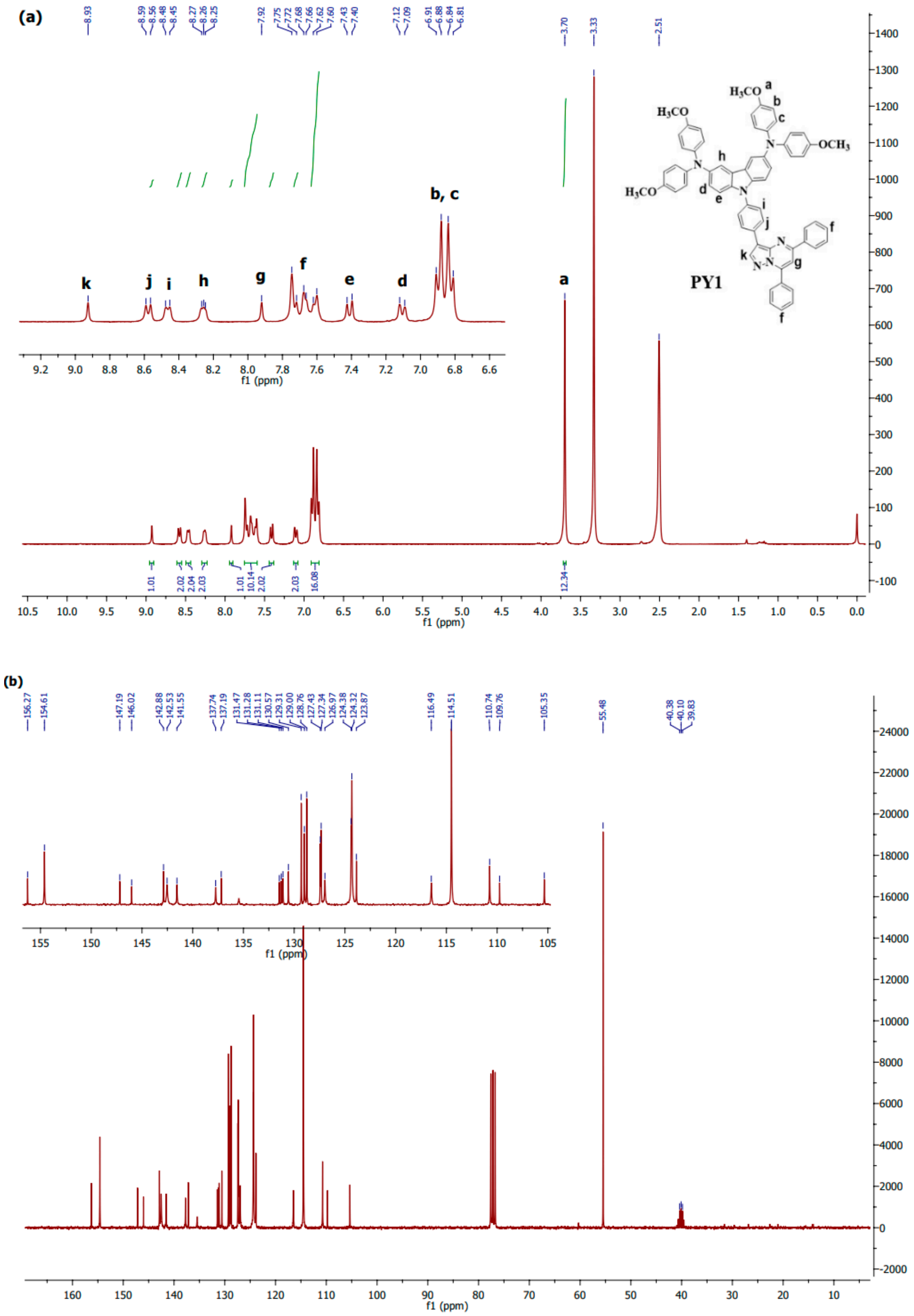

Figure S3. NMR spectrum of **PY1**. (a) ^{1}H NMR spectrum in DMSO-d_6 and (b) ^{13}C NMR spectrum in $CDCl_3$/DMSO-d_6.

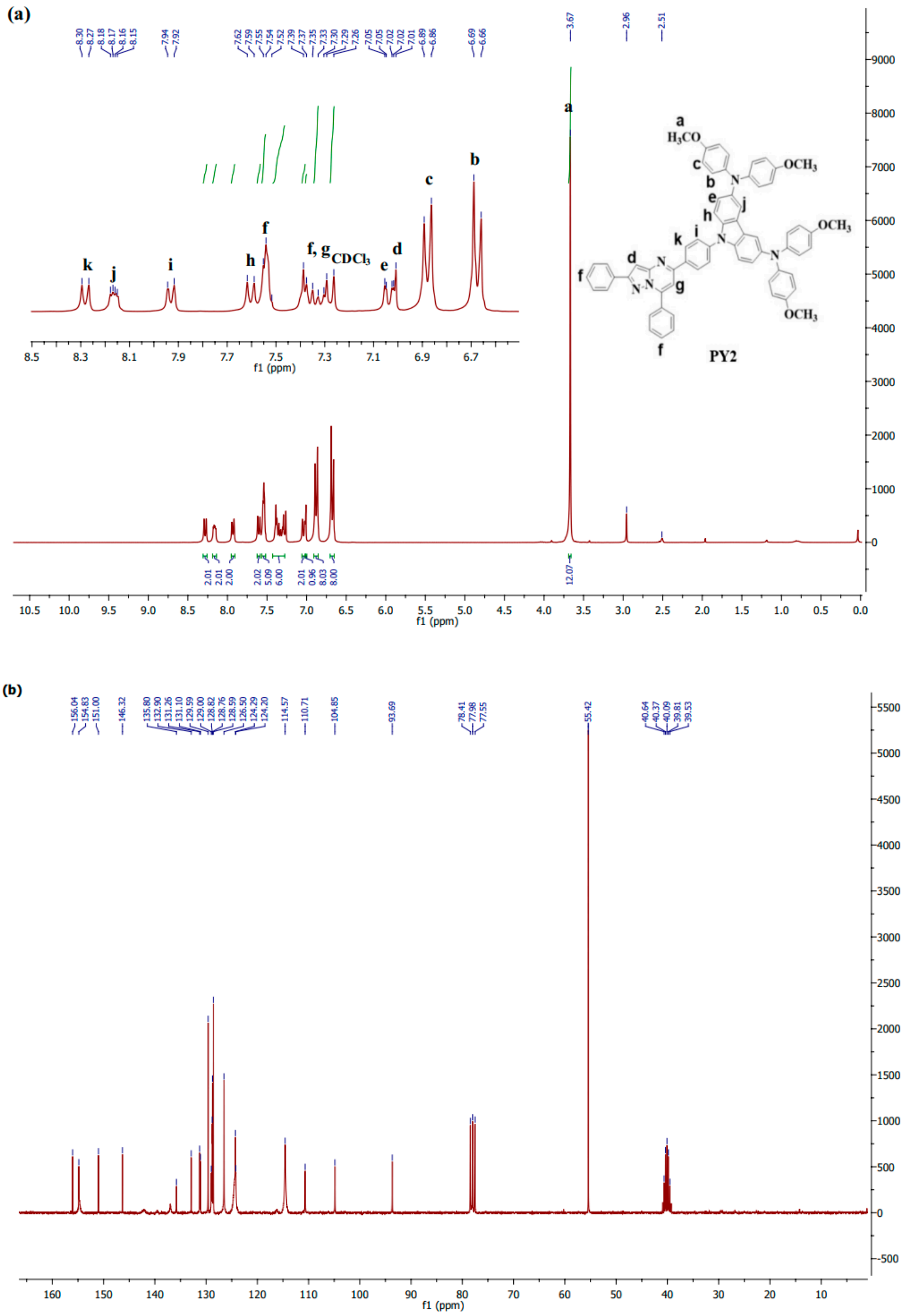

Figure S4. NMR spectrum of **PY2**. (a) ^{1}H NMR spectrum and (b) ^{13}C NMR spectrum in $CDCl_3$/DMSO-d_6.

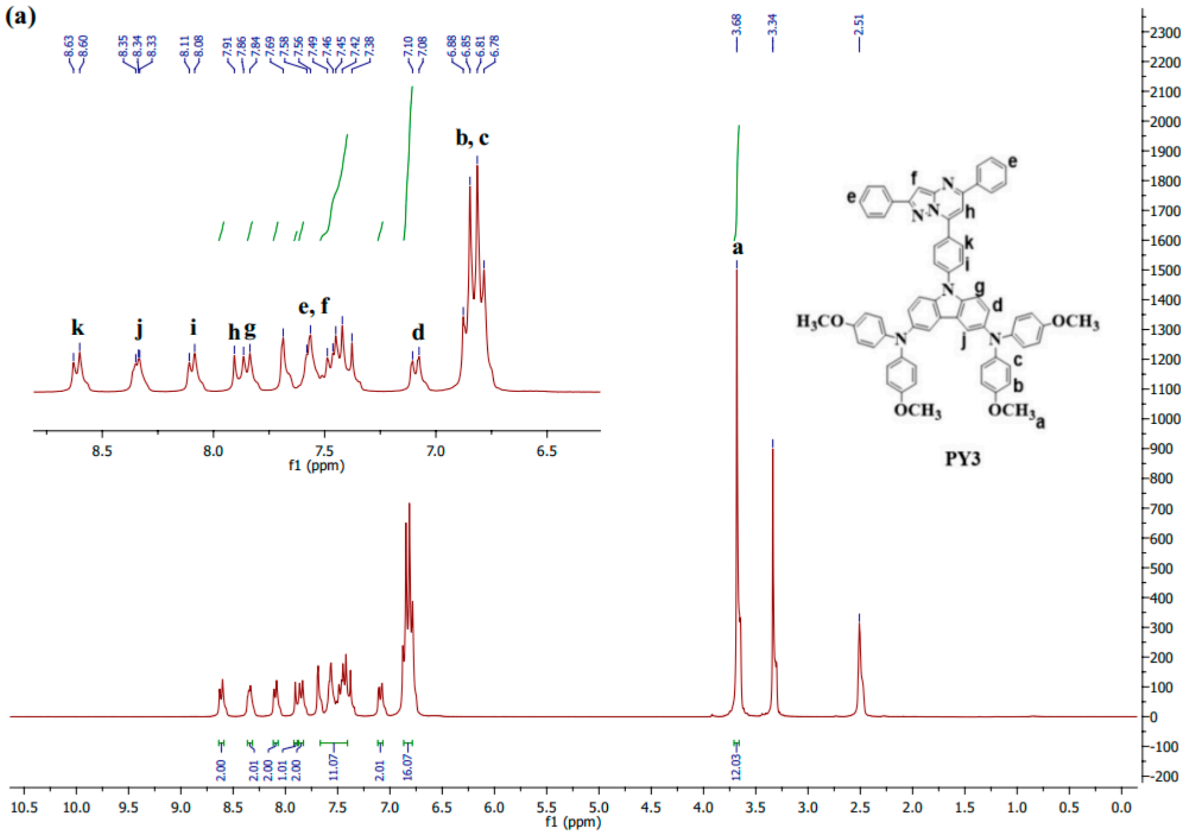

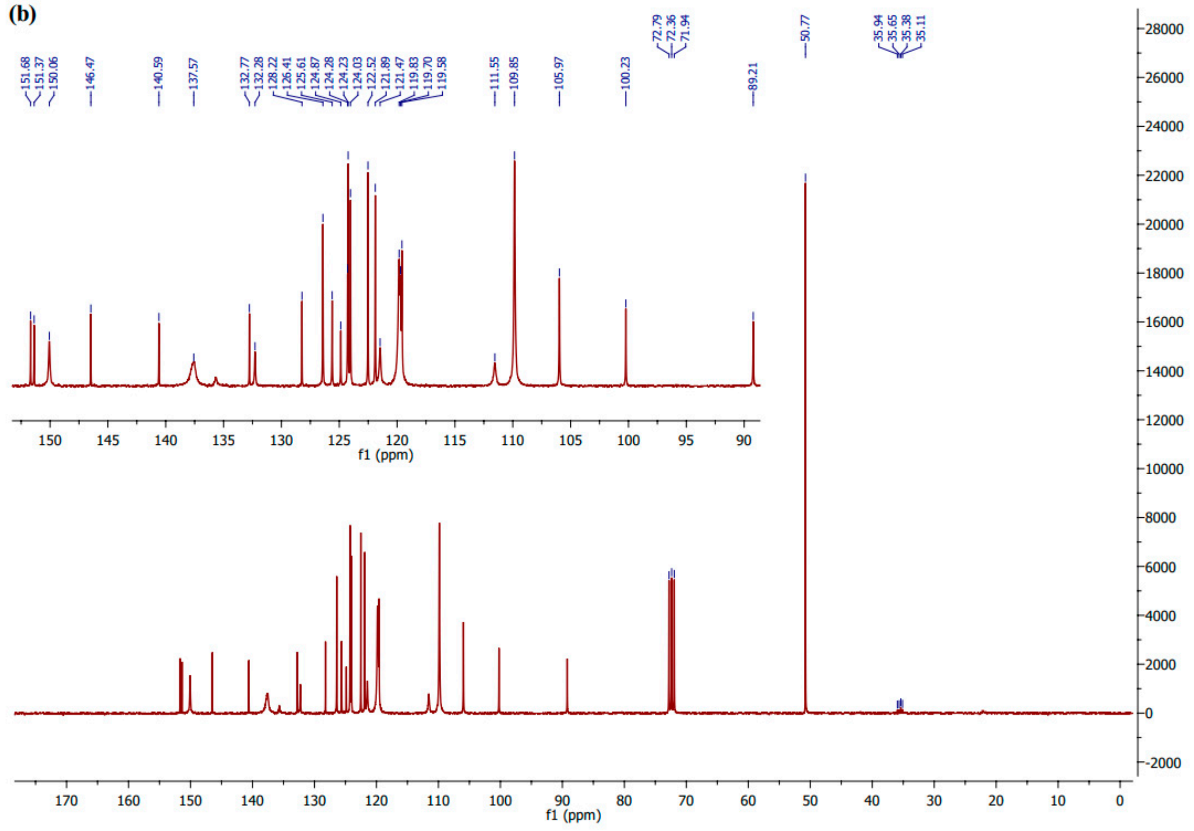

Figure S5. NMR spectrum of **PY3**. (a) ^{1}H NMR spectrum in DMSO-d_6 and (b) ^{13}C NMR spectrum in $CDCl_3$/DMSO-d_6.

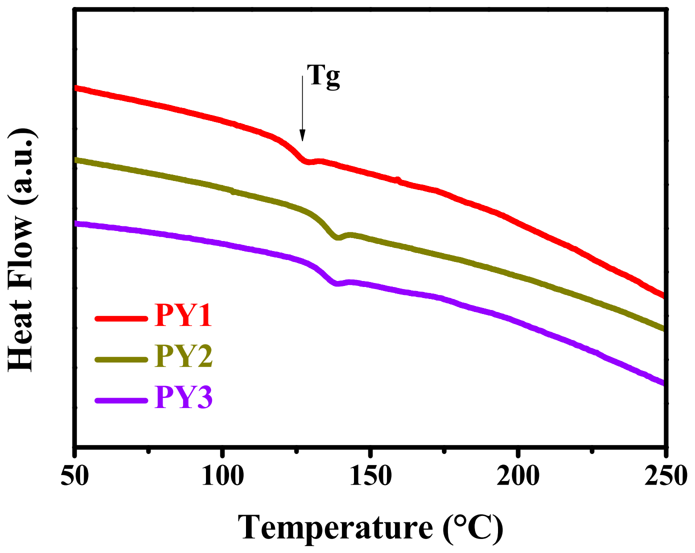

Figure S6. DSC traces of the three HTMs (second heating) at a scan rate of 5°C/min under N_2 atmosphere.

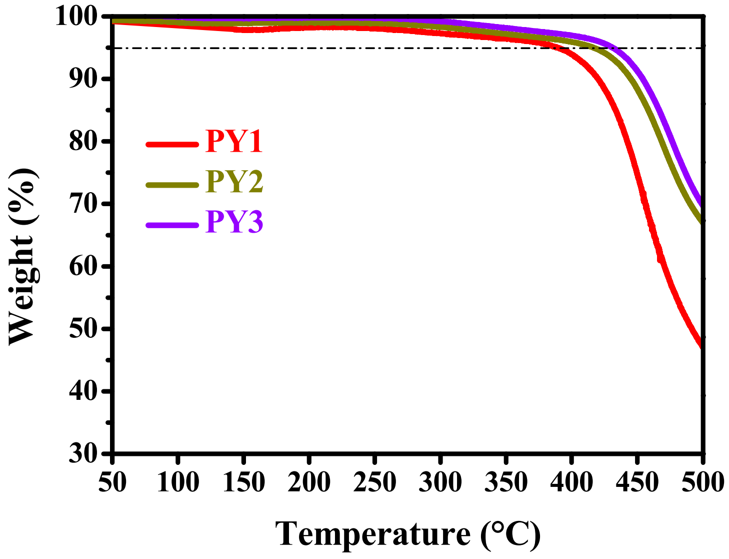

Figure S7. TGA curves of the three HTMs at a heating rate of 10°C/min under N_2 atmosphere.

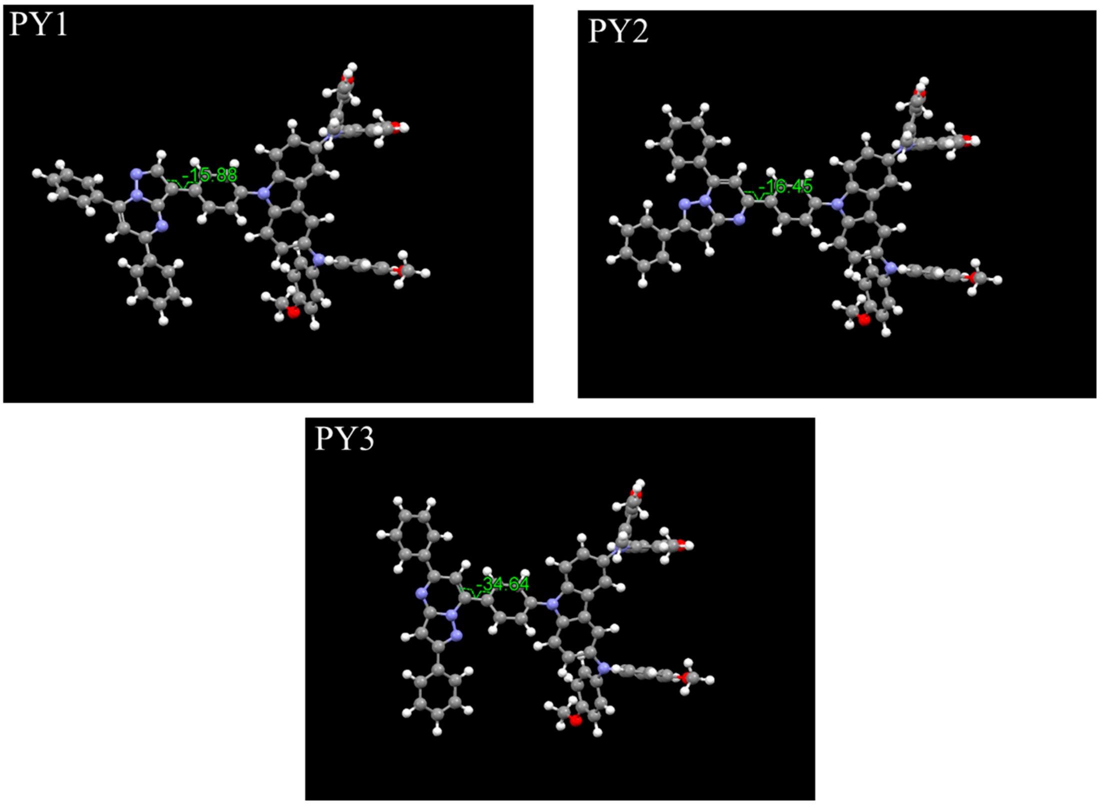

Figure S8. Geometry optimized structures of **PY1–PY3** and dihedral angles between phenyl spacer and pyrazolo[1,5-a]pyrimidine acceptor core, calculated by DFT at the B3LYP/6-31G level.

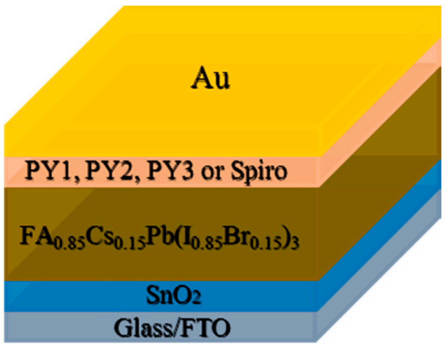

Figure S9. Schematic of the device configuration used.

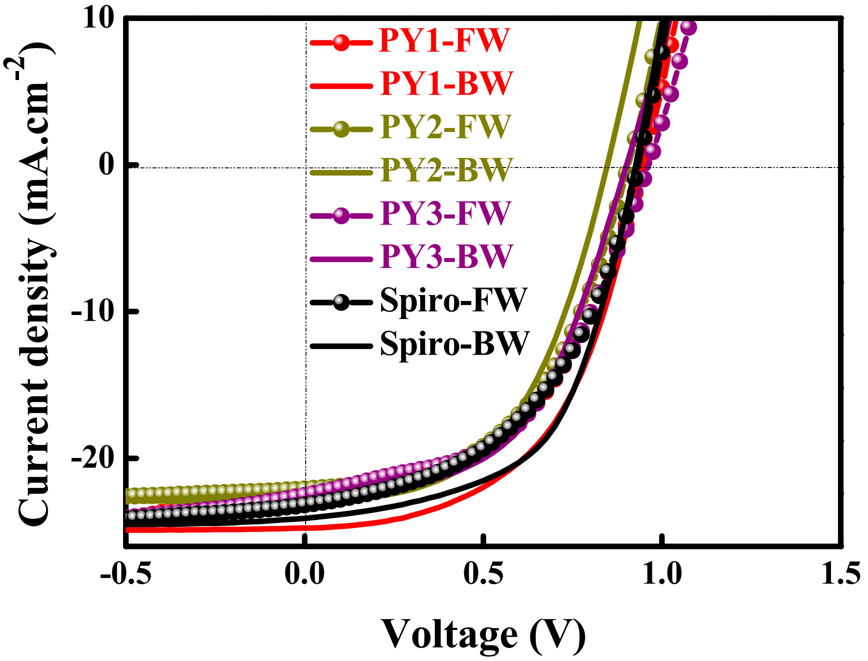

Figure S10. J-V curves of the best PSCs using different HTMs under simulated AM1.5G illumination (forward and backward).

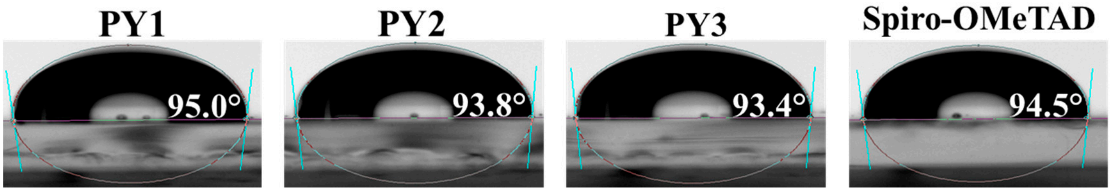

Figure S11. Water-contact angles measurement of the three HTMs and Spiro-OMeTAD films deposited on glass substrates.
